# Experimental Evaluation of the Protective Efficacy of Tick-Borne Encephalitis (TBE) Vaccines Based on European and Far-Eastern TBEV Strains in Mice and *in Vitro*

**DOI:** 10.3389/fmicb.2018.01487

**Published:** 2018-07-16

**Authors:** Liubov L. Chernokhaeva, Yulia V. Rogova, Liubov I. Kozlovskaya, Lidiya I. Romanova, Dmitry I. Osolodkin, Mikhail F. Vorovitch, Galina G. Karganova

**Affiliations:** ^1^Chumakov Institute of Poliomyelitis and Viral Encephalitides, Chumakov Federal Scientific Center for Research and Development of Immune-and-Biological Products Russian Academy of Science, Chumakov FSC R&D IBP RAS, Moscow, Russia; ^2^Institute for Translational Medicine and Biotechnology, Sechenov First Moscow State Medical University, Moscow, Russia; ^3^Department of Chemistry, Lomonosov Moscow State University, Moscow, Russia; ^4^Department of Biology, Lomonosov Moscow State University, Moscow, Russia

**Keywords:** TBE vaccine, antibody, TBEV, immune response, E protein, antigen determinants

## Abstract

Tick-borne encephalitis (TBE), caused by the TBE virus (TBEV), is a serious public health threat in northern Eurasia. Three subtypes of TBEV are distinguished. Inactivated vaccines are available for TBE prophylaxis, and their efficacy to prevent the disease has been demonstrated by years of implication. Nevertheless, rare TBE cases among the vaccinated have been registered. The present study aimed to evaluate the protective efficacy of 4 TBEV vaccines against naturally circulating TBEV variants. For the first time, the protection was evaluated against an extended number of phylogenetically distinct TBEV strains isolated in different years in different territories. The protective effect did not strongly depend on the infectious dose of the challenge virus or the scheme of vaccination. All vaccines induced neutralizing antibodies in protective titers against the TBEV strains used, although the vaccines varied in the spectra of induced antibodies and protective efficacy. The protective efficacy of the vaccines depended on the individual properties of the vaccine strain and the challenge virus, rather than on the subtypes. The neutralization efficiency appeared to be dependent not only on the presence of antibodies to particular epitopes and the amino acid composition of the virion surface but also on the intrinsic properties of the challenge virus E protein structure.

## Introduction

Tick-borne encephalitis (TBE) poses a serious public health concern in Europe and Northern Asia. Annually, 10,000–12,000 clinical TBE cases are reported worldwide, and the incidence appears to be increasing (Kollaritsch et al., 2011). In Russia alone, over 60 million people live in TBE endemic territories (Chernokhaeva et al., 2016a).

The disease is caused by the tick-borne encephalitis virus (TBEV). Three subtypes of TBEV are distinguished: Far-Eastern (FE), Siberian (Sib), and European (Eur) (King et al., 2012), and their distribution largely, but not completely, corresponds to their names. Moreover, an expansion of the Sib TBEV subtype toward Northern Europe has been observed (Jääskeläinen et al., 2016). All three subtypes (Golovljova et al., 2004) were found in Baltic countries and in Russia. Recently, new phylogenetic groups, including Transbaikal (TB) and Buryat-Mongol (BM) ones, have been described in Siberia and Mongolia (Demina et al., 2010; Khasnatinov et al., 2010). All described TBEV variants co-circulate in Russia (Zlobin et al., 2007; Demina et al., 2010).

Preventive vaccination is a primary prophylactic tool against TBE. Inactivated, purified, concentrated vaccines for adults and children based on FE strains Sofjin (Elbert et al., 1980; Vorovitch et al., 2015), 205 (Safronov et al., 1991; Karpova et al., 1995), and Senzhang (Zhang et al., 2012; Xing et al., 2017) and on Eur strains Neudoerfl (Ecker et al., 1999; Loew-Baselli et al., 2011) and K23 (Ecker et al., 1999; Zent et al., 2003) are available. Clinical trials have demonstrated their immunogenicity (Vorob'eva et al., 1983; Pavlova et al., 1999; Amicizia et al., 2013; Vorovitch et al., 2017). The significant decrease in TBE incidence in regions with a vaccination coverage of >80% of the population also confirms the high protective efficacy of the TBE vaccines in the territories where the subtype of the vaccine strain corresponds to the subtype of the circulating virus (Heinz et al., 2007; Heinz and Stiasny, 2012) or differs from it (Romanenko et al., 2006, 2007; Kovalev et al., 2008).

Nevertheless, TBE cases among completely vaccinated subjects have been reported (Romanenko et al., 2007; Andersson et al., 2010; Grgič-Vitek et al., 2010) in different age groups, with a peak in adults over 50 (Andersson et al., 2010; Grgič-Vitek et al., 2010). In most cases, TBE has manifested as a mild fever. However, isolated cases of severe disease forms and fatal outcomes have been registered among vaccinated patients (Andersson et al., 2010; Pogodina et al., 2015). The causes and conditions of these cases remain unclear. Insufficient protection of the vaccinated person against TBEV infection can be related to their compromised status (genetic predisposition, immune status, concomitant infections, etc.) or to virus-related features (infectious dose, virus properties, etc.) In light of this fact, an evaluation of the sufficiency of the vaccine-induced immune response for protection from all naturally circulating TBEV variants seems to be of crucial importance.

The use of a set of TBEV strains in the neutralization test with sera of vaccinated subjects provides indirect information on the degree of protection from different virus variants. Previously, such experiments showed that vaccines based on Eur and FE strains induce neutralizing antibodies (nAbs) against all 3 TBEV subtypes (Amicizia et al., 2013; Domnich et al., 2014; Maikova et al., 2016) and even other flaviviruses (Clarke, 1964; Calisher et al., 1989; Pripuzova et al., 2013; McAuley et al., 2017). However, it seems that the presence of nAbs in the sera of immunized subjects does not always reliably reflect the degree of protection (Chernokhaeva et al., 2016b), especially when a heterologous virus is used for a challenge (Pripuzova et al., 2013).

Animal-model experiments provide more detailed information on vaccine protective efficacy. A correlation between the results of tests in mice and vaccine immunogenicity in humans has been previously reported for a vaccine based on the Sofjin strain (Elbert et al., 1981). It has been shown for a limited number of TBEV strains that existing vaccines effectively protect laboratory mice from TBEV strains of different subtypes (Holzmann et al., 1992; Leonova and Pavlenko, 2009; Morozova et al., 2014). Similar results were obtained with the use of recombinant viruses carrying protein E sequences of three TBEV strains (Fritz et al., 2012).

We recently described the spectrum of antiviral nAbs and protective immunity induced by the vaccine based on FE strain Sofjin against 14 TBEV strains isolated in different years in different regions and representing different phylogenetic lineages of TBEV (Chernokhaeva et al., 2016b). The vaccine protected against all TBEV strains used; the protective efficacy was similar for the homologous and heterologous virus variants used for challenge.

In the present study, we evaluated the impact of individual properties of the vaccine strains and the challenge viruses on the vaccine-induced immune response in experiments *in vitro* (plaque neutralization test) and *in vivo* (experiments in mice). We used vaccines based on FE and Eur TBEV strains and a wide range of TBEV strains compared in extreme conditions (most distinct vaccine and challenge strains, high doses of a challenge virus) by protective efficacy in mice and spectra of nAbs, and we attempted to tie the differences to E protein structures.

## Materials and methods

### Cells and viruses

Porcine embryo kidney (PEK) cells were maintained on 199 medium with 5% fetal bovine serum (Gibco) at 37°C (Kozlovskaya et al., 2010).

TBEV strains (Table 1) were described previously (Gritsun et al., 1997; Romanova et al., 2007; Zlobin et al., 2007; Kozlovskaya et al., 2010; Vorovitch et al., 2015; Chernokhaeva et al., 2016b; Shevtsova et al., 2016). The viruses were stored at −70°C as a 10% mouse brain suspension or a culture supernate (CS) of infected PEK cells.

**Table 1:** TBEV strains used in the study.

**TBEV strain**	**Region and year of isolation**	**Origin of isolation**	Passages^*^	**GenBank accession #**	**References**
**FAR-EASTERN SUBTYPE**					
Sofjin/Sofjin-KGG	Primorskiy krai, Russia, 1937	brain of a TBE patient	MxM2	KC806252, GU121963	Kozlovskaya et al., 2010; Vorovitch et al., 2015
205/205KGG	Khabarovskiy krai, Russia, 1973	*I. persulcatus*	MxM1P3	DQ989336, GU121964	Safronov et al., 1991; Kozlovskaya et al., 2010
DV936k	Primorskiy krai, Russia, 1975	*I. persulcatus*	M3P2	GU125722	Kozlovskaya et al., 2010; Chernokhaeva et al., 2016b
**EUROPEAN SUBTYPE**					
Absettarov	Leningrad region, Russia, 1951	blood of a TBE patient	MxM5	KU885457	Kozlovskaya et al., 2010; Shevtsova et al., 2016
LK-138	Lithuania, 1972	*I. ricinus*	MxM1P1	GU125720	Kozlovskaya et al., 2010; Chernokhaeva et al., 2016b
**SIBERIAN SUBTYPE**					
Vasilchenko	Novosibirsk region, Russia, 1961	blood of a TBE patient	MxM3	L40361	Gritsun et al., 1997; Chernokhaeva et al., 2016b
YuK 4/13	Kemerovo region, Russia, 1969	*I. persulcatus*	M4P2M1	GU125721	Kozlovskaya et al., 2010; Chernokhaeva et al., 2016b
EK-328	Estonia, 1971	*I. persulcatus*	M6P1M4	DQ486861	Romanova et al., 2007; Shevtsova et al., 2016
Lesopark 11	Novosibirsk, Russia, 1986	*I. persulcatus*	MxM2	GU121966	Kozlovskaya et al., 2010; Morozova et al., 2014
TV08-T2546	Republic of Tuva, Russia, 2008	*I. persulcatus*	M2P1	KU052690	Chernokhaeva et al., 2016b
Karl08-T3522	Republic of Karelia, Russia, 2008	*I. persulcatus*	M3P1	KU052689	Chernokhaeva et al., 2016b
**TRANSBAIKAL GROUP**					
178-79	Irkutsk region, Russia, 1979	*I. persulcatus*	MxM1	EF469661	Zlobin et al., 2007; Khasnatinov et al., 2010
**BURYAT-MONGOL GROUP**					
886-84	Irkutsk region, Russia, 1984	Brain of *Clethrionomys rufocanus*	MxM1	EF469662	Zlobin et al., 2007; Khasnatinov et al., 2010

* *M, passages in mouse brain (Mx, passages performed by strain authors before the viruses were obtained in the laboratory); P, passages in PEK cells*.

### Vaccines

We used a cultural, purified, concentrated, inactivated, lyophilized TBE (Moscow) vaccine based on the Sofjin strain (Chumakov PIPVE, now Chumakov FSC R&D IBP RAS, Russia); EnceVir, based on strain 205 (Virion Company, Microgen, Russia); FSME-Immun Inject (FSME), based on strain Neudoerfl (Baxter Vaccine AG, now Pfizer, Austria); and Encepur Adult vaccine, based on strain K23 (Chiron Bering GmbH & Co., now Novartis, Germany). The E protein content in a vaccine dose is 0.5–0.75 μg for the Moscow vaccine, 2.0–2.5 μg for EnceVir, 2.4 μg for FSME, and 1.5 μg for Encepur (Kollaritsch et al., 2011).

### Ethics statement

Mice were maintained according to international guidelines for animal husbandry and Chumakov FSC R&D IBP RAS ethical guidelines. Experiments were approved by the Chumakov FSC R&D IBP RAS ethics committee.

All experiments were performed in the BSL-2 and−3 facilities, as prescribed by the institutional and national guidelines.

### Evaluation of *in vivo* protective efficacy of the vaccines

Eight-week-old BALB/c mice (Scientific Center of Biomedical Technologies, Stolbovaya branch, Russia) were injected intramuscularly (upper third of the thigh) with the studied vaccines (1/10 human dose with 2–4 weeks between immunizations, specified in the Results). Two/four weeks post-immunization mice were subcutaneously (s/c) infected with the virus (the terms and doses are specified in the Results). Vaccination and challenge schemes reflected the possible real-life situations, which were specified in the manufacturer's instructions as standard and emergent (rapid, accelerated) schemes. The mice were monitored daily for 21 days post infection (d.p.i.), and clinical outcomes were classified as follows: *m* = 1 if the mice were untidy, clumsy, or lost weight over 1.5 g for at least 3 days; and *m* = 2 if the mice showed signs of intoxication, paresis and paralysis of limbs. Each experiment included a group of mice for virus titration (LD50) to estimate and control the exact dose of challenge virus.

### 50% plaque reduction neutralization test (PRNT50)

Twenty-seven mice were intramuscularly immunized twice with a 1/10 human dose with a 30-day interval. Blood was taken 14 days after the second immunization. The sera of mice immunized with the same vaccine were pooled, inactivated at 56°C for 30 min, aliquoted, and stored at −20°C. PRNT50 was performed on PEK cells as described previously (Pripuzova et al., 2009).

### Statistical analysis

In the mouse experiments, a statistical evaluation was performed with the Fisher exact test (FET). Geometric mean titers (GMTs) of the nAbs and variances were calculated.

### Sequence alignment and protein structure visualization

An amino acid sequence alignment was built manually and analyzed and rendered in Jalview 2.8 (Waterhouse et al., 2009). The protein structure was visualized in VMD 1.9.1 (Humphrey et al., 1996).

## Results

### Dose of challenge virus inoculation and the protective efficacy of TBE vaccine based on eur strain

We evaluated the effect of the challenge virus dose on the protective efficacy of the FSME vaccine based on Eur strain Neudoerfl. Sib strain Lesopark 11 was used as a heterologous challenge virus.

The animals were protected from death and disease against a wide range of doses of the challenge virus (Table 2). No correlations were found between vaccine effectiveness and virus dose in the specified dose range (the differences were insignificant, FET). Nevertheless, even at the low challenge virus dose, fewer than 100% of the mice were protected, and <50% of the animals were protected from the disease at all doses.

**Table 2:** Effect of challenge-virus dose (Sib TBEV strain Lesopark 11) on protective activity of FSME vaccine, based on Eur TBEV strain.

**Virus dose, LD50**	**Immunization**	**Number of animals**	**Survived animals, %**	**Healthy animals, %**	**Surviving mice with clinical signs, %^b^**	
					**Mild (*m* = 1)**	**Severe (*m* = 2)**
1.3^*^	+^a^	7	71	42	29	0
	–	7	14	0	0	0
13	+	7	57	43	14	0
	–	7	0	0	0	0
130	+	7	57	43	0	14
	–	7	0	0	0	0
1,300^*^	+	7	86	43	29	14
	–	7	0	0	0	0
13,000^*^	+	7	86	43	43	0
	–	7	14	0	0	14

*Mice were immunized twice with 4 weeks interval, and challenged 2 weeks after the second immunization*.

a *“+” immunized group, “–” control group, without immunization*.

b *m = 1, mice were untidy, clumsy, and lost weight over 1.5g for at least 3 days; m = 2, mice showed signs of intoxication, paresis and paralysis of limbs*.

* *Protective activities in these groups are statistically significant (the difference between immunized animals and control group)*.

### Spectrum of protective efficacy of the FSME vaccine *in vivo*

The spectrum of protective efficacy of the vaccine based on the Eur strain was evaluated using a set of TBEV strains belonging to different subtypes *in vivo*. The results are summarized in Table 3.

**Table 3:** Spectrum of protective activity of FSME vaccine *in vivo*.

**Strain**	**Virus dose, LD50**	**Immunization**	**Number of animals**	**Survived animals, %**	**Healthy animals, %**	**Surviving mice with clinical signs, %^b^**	
						**Mild (*m* = 1)**	**Severe (*m* = 2)**
**EUROPEAN SUBTYPE**							
Absettarov	230	+^a^	10	100	100	0	0
		–	11	0	0	0	0
LK-138	4700	+	15	80	60	7	13
		–	15	0	0	0	0
**FAR-EASTERN SUBTYPE**							
SofjinKGG	200	+	15	27	0	20	7
		–	16	0	0	0	0
205KGG	70	+	11	100	82	18	0
		–	9	0	0	0	0
**SIBERIAN SUBTYPE**							
YuK4/13	2	+	11	100	82	9	9
		–	10	10	0	10	0
TV08-T2546	6	+	11	90	45	45	0
		–	10	60	10	30	20

*Mice were immunized twice with 4 weeks interval, and challenged 4 weeks (2 weeks for LK-138) after the second immunization*.

a *“+” immunized group, “–” control group, without immunization*.

b *m = 1, mice were untidy, clumsy, and lost weight over 1.5g for at least 3 days; m = 2, mice showed signs of intoxication, paresis and paralysis of limbs*.

The vaccine effectively protected 80–100% of the animals against TBEV strains belonging to the Eur subtype, even against a high challenge virus dose (4,700 LD50). FSME-immunized mice were sufficiently protected against low doses of the Sib TBEV strains. However, mild disease signs were observed in almost 50% of immunized animals s/c challenged with 6 LD50 of Sib strain TV08-T2546. Vaccination provided complete protection against FE strain 205; nevertheless, 18% of the surviving animals had clinical signs. In the case of FE strain Sofjin as the challenge virus, 73% of the immunized animals died, and all surviving animals had disease signs.

### Comparison of the protective efficacy of different vaccines in the same experiment

The protective efficacy of different TBE vaccines against the set of TBEV strains was compared in the same experiment to minimize the influence of non-control parameters (Table 4).

**Table 4:** Protective activity in mice of TBE vaccines against a set of TBEV strains.

**Strain**	**Virus dose, LD50**	**Vaccine**	**Number of animals**	**Survived animals, %**	**Healthy animals, %**	**Surviving mice with clinical signs, %^b^**	
						**mild (*m* = 1)**	**Severe (*m* = 2)**
INTERVALS BETWEEN IMMUNIZATIONS AND CHALLENGE, 0^*^4^*^2 WEEKS							
**European Subtype**							
Absettarov	1,800	**Moscow**	15	87	87	0	0
		**EnceVir**	15	100	100	0	0
		Encepur	15	80	80	0	0
		–^a^	15	7	7	0	0
	3,200	**Moscow**	16	100	100	0	0
		**EnceVir**	15	100	100	0	0
		FSME	15	100	100	0	0
		–	15	0	0	0	0
**Far-eastern Subtype**							
Sofjin	400	**Moscow**	15	87	87	0	0
		**EnceVir**	15	87	87	0	0
		Encepur	15	27	27	0	0
		–	15	0	0	0	0
**Siberian Subtype**							
EK-328	1,400	**Moscow**	14	86	86	0	0
		**EnceVir**	13	92	92	0	0
		Encepur	16	75	56	6	13
		–	15	7	7	0	0
**Transbaikal Group**							
178-79	420	**Moscow**	21	100	100	0	0
		FSME	10	90	90	0	0
		–	10	0	0	0	0
**Buryat-mongol Group**							
886-84	230	**Moscow**	15	100	100	0	0
		FSME	15	47	40	0	7
		–	15	0	0	0	0
INTERVALS BETWEEN IMMUNIZATIONS AND CHALLENGE, 0^*^4^*^4 WEEKS							
**European subtype**							
LK-138	600	**Moscow**	13	100	92	8	0
		FSME	11	64	55	9	0
		–	12	0	0	0	0
**Far-Eastern subtype**							
DV936k	4000	**Moscow**	11	100	73	9	18
		FSME	11	91	55	27	9
		–	10	10	0	0	10
**Siberian subtype**							
EK-328	1000	**Moscow**	16	88	88	0	0
		Encepur	15	54	47	0	7
		FSME	18	67	56	11	0
		–	18	12	6	6	0

a *“–” control group without immunization*.

b *m = 1, mice were untidy, clumsy, and lost weight over 1.5g for at least 3 days; m = 2, mice showed signs of intoxication, paresis and paralysis of limbs*.

*Vaccines based on strains of the Far-Eastern subtypes are shown in bold*.

*All the vaccines were tested against each strain in one experiment*.

All vaccines provided a high level of protection (80–100%) from high doses of Eur strain Absettarov. Immunization with the Moscow or FSME vaccines reliably (*P* < 0.05, FET) protected the mice from the high dose of Eur strain LK-138. Therefore, the FSME vaccine ensured significantly (*P* = 0.03, FET) better protection from the higher dose of Absettarov strain than from the LK-138 strain, whereas the Moscow vaccine demonstrated total protection from both strains.

The Moscow and FSME vaccines protected the animals after a challenge with a high dose of FE strain DV936. Vaccines based on FE strains (Moscow and EnceVir) provided a high level of protection from the Sofjin strain. Encepur based on the Eur strain did not protect mice from this strain (*P* = 0.10, FET).

All four vaccines protected the animals from Sib strain EK-328 (*P* < 0.02, FET). Neither for the Moscow vaccine nor for Encepur was the level of protection affected by changes in the time of challenge (2 weeks after the last immunization against four).

The FSME and Moscow vaccines provided almost complete protection against strain 178-79 from a new TB group. FSME vaccine protected almost 50% animals against the prototype 886-84 strain of the new MB group, but ensured significantly (*P* = 0.002, FET) lower protection than the Moscow vaccine did.

### Evaluation of immunogenicity of TBEV vaccines in the PRNT50

In PRNT50, we studied pooled sera against a wider set of TBEV strains with each virus strain in the same experiment (Table 5).

**Table 5:** Titers of neutralizing antibodies (nAb) in pooled sera mice immunized with different vaccines (log_10_).

**Virus**	**Vaccine**	**Titres of nAb induced by the vaccines, log**_**10**_					**Variance 2 (σ_2_)**
		**Based on FE strains**		**Based on Eur strains**		**GMT_2_, log_10_**	
**Subtype**	**Strain**	**Moscow**	**EnceVir**	**FSME**	**Encepur**		
FE	SofjinKGG	2.12	1.60	1.54	1.95	1.80	0.077
	205KGG	1.80	2.00	2.05	0.90	1.69	0.287
	DV936	1.86	2.80	1.77	2.38	2.20	0.231
Eur	Absettarov	1.36	1.72	1.98	2.08	1.78	0.103
	LK-138	1.79	1.24	2.08	2.40	1.88	0.243
Sib	EK-328	1.82	2.22	2.88	2.83	2.44	0.259
	Lesopark 11	1.98	2.98	2.37	2.39	2.43	0.170
	Vasilchenko	1.41	1.60	1.25	1.82	1.52	0.060
	YuK4/13	2.20	1.18	1.32	2.54	1.81	0.441
	KarlT08-3522	1.66	2.76	1.00	1.73	1.79	0.529
	GMT_Sib_, log_10_	1.71	1.94	2.06	1.75		
	Variance Sib (σ_Sib_)	0.10	0.48	0.64	0.22		
TB	178-79	1.60	1.28	1.80	2.20	1.72	0.148
BM	886-84	1.76	2.35	1.86	1.87	1.96	0.070
	GMT_1_, log_10_	1.78	1.98	1.82	2.09		
	Variance 1 (σ_1_)	0.064	0.410	0.264	0.247		
							
		0.90				2.98	
		Titres of nAb, log_10_					

*Mice in groups of 27 per each vaccine were immunized twice with 4 weeks interval, and serum samples were collected 2 weeks after the second immunization*.

Pooled sera from mice immunized with all tested vaccines contained nAbs against almost all TBEV strains used in a protective titer (>1:10). The highest nAb titers were not found against the vaccine strain or strain of the corresponding TBEV subtype. For instance, immunization with Encepur and FSME induced nAbs in the highest titers to Sib strain EK-328 (2.83 and 2.88 log_10_, respectively). EnceVir induced the highest titers of nAbs against Sib strain Lesopark 11 (2.98 log_10_). These findings suggest that the PRNT50 results depend not only on the relationship between the antigen similarity of the viruses used for immunization and for PRNT50 but also on other characteristics of the virus sample.

**Figure 1:**
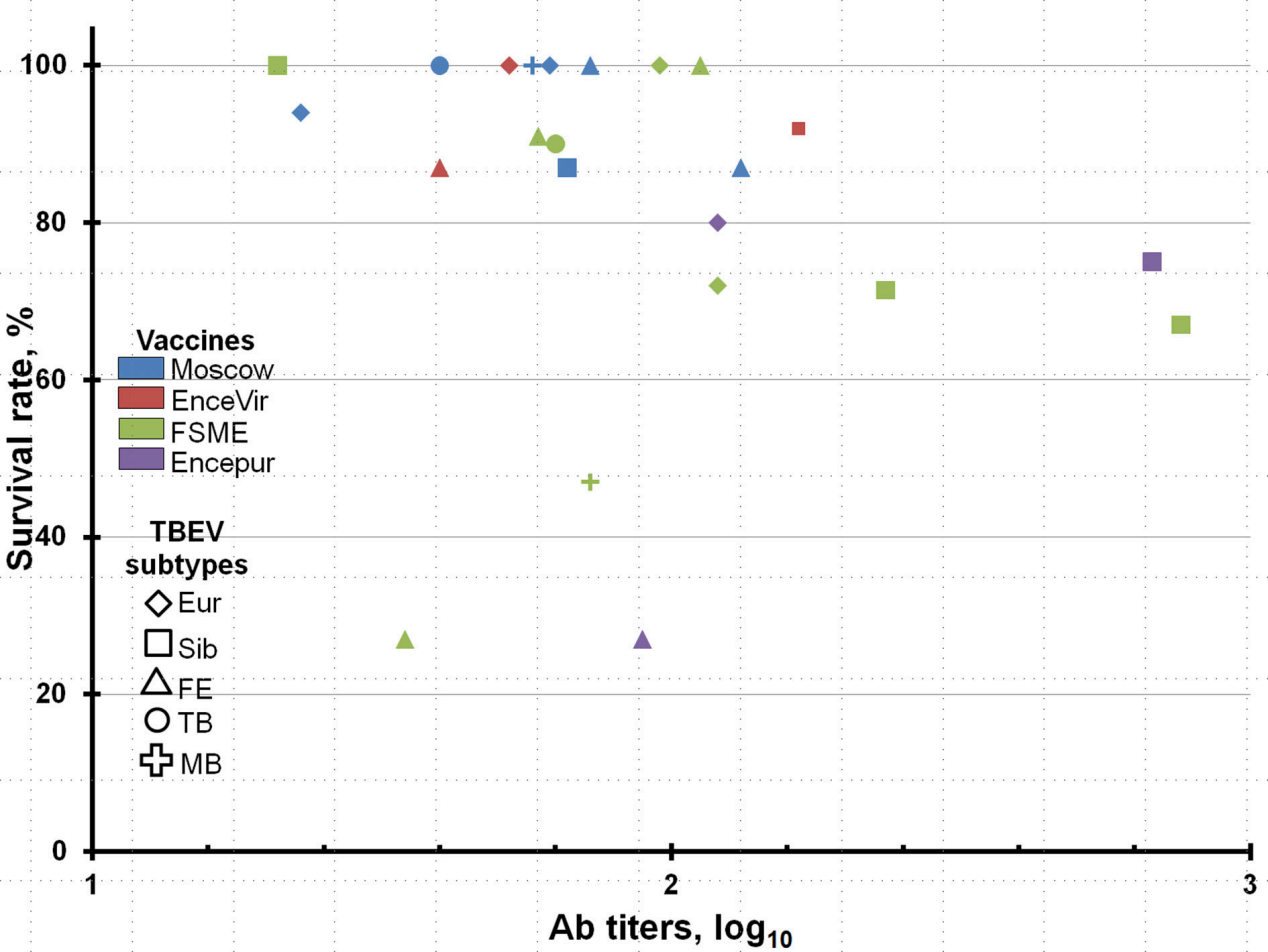
Correlations between neutralizing antibody titers and protectivity (survival rates) in animals, immunized with various vaccines, against studied TBEV strains.

In most cases, a high level of protection (80–100%) was observed at titers of nAbs >1 log_10_ (Tables 2–5, Figure 1). However, in some cases, despite the high titers of nAbs, the protective efficacy was below 80%, as follows: for the FSME vaccine against strains 886-84 (47% of the animals survived), EK-328 (67%), and LK-138 (75%, mean for two experiments); and for the Encepur vaccine against strains EK-328 (67%, mean for two experiments) and Sofjin (27%). This finding suggests that the protective effect of the vaccine against a specific strain depends not only on the spectrum of nAbs but also on other characteristics of the particular challenge virus strain.

### Analysis of the spectrum of nAbs and assessment of the antigenic relationships between vaccine and TBEV strains *in vitro*

The GMTs of nAbs in pooled mouse sera against 12 TBEV strains (3 FE, 2 Eur, 5 Sib, and 2 strains, representing two new phylogenetic groups, TB and BM) varied from 1.78 log_10_ for the Moscow vaccine to 2.09 log_10_ for the Encepur vaccine (Table 5). The differences were statistically insignificant.

We used the variance of nAb titers induced by a particular vaccine against the set of TBEV strains (Table 5, Variance 1, σ_1_) to estimate the breadth of the nAb spectrum in the sera; a smaller difference between nAb titers to different strains (σ_1_) means a wider nAb spectrum. The variance was minimal (0.064) for the Moscow vaccine, while other vaccines demonstrated a similar variance (0.247–0.410). This indicated that the sera of mice immunized with the Moscow vaccine contained nAbs to all the studied TBEV strains in similar titers, while sera from mice immunized with other vaccines contained high titers of nAbs against one strain and low titers against another one.

Vaccines based on the FE and Eur TBEV strains induced a pronounced immune response against TBEV strains of the Sib subtype. Titers of nAbs against 5 strains of this subtype in mouse sera varied from 1.71 log_10_ for the Moscow vaccine to 2.06 log_10_ for the Encepur vaccine. Again, the Moscow vaccine showed the lowest variance (Table 5, variance Sib, σ_Sib_) (0.102), and the FSME vaccine showed the highest variance (0.639).

The differences in the nAb titers induced by the vaccines against a particular strain to a certain extent reflect the antigenic similarity between the vaccine strain and virus used in the PRNT50. We used the variance of nAb titers against a particular strain (Table 5, variance 2, σ_2_) to estimate the ability of the strain to escape the vaccine-induced immune response. The variance considerably varied from strain to strain. Minimal variance (0.060) was noted for strain Vasilchenko. The maximum variance (0.528) in this assay was demonstrated for the strain Karl08-T3522.

### Analysis of a correlation between the antigenic differences in the PRNT50 and the structures of protein E of TBEV strains

**Figure 2:**
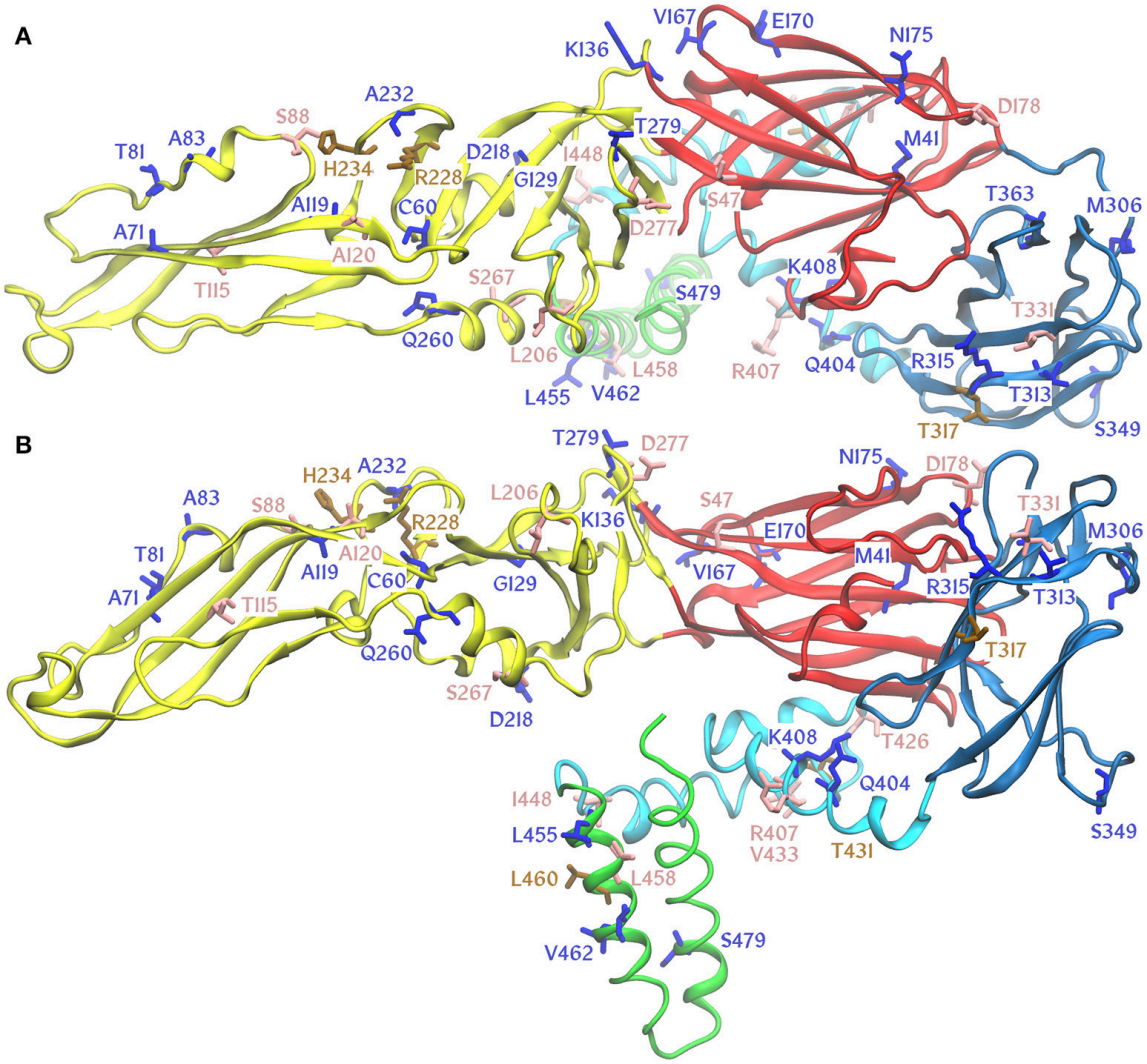
Amino acid substitutions in TBEV E protein differing TBE vaccine strains and studied viruses mapped onto a homology model of E protein from EK-328 strain [Osolodkin, manuscript in preparation]. Protein domains are marked by color: I, red; II, yellow; III, blue; stem, cyan; anchor, green. Substitutions are marked by color: unique, blue; subtype specific, salmon; variable positions, brown. **(A)**, view from the top; **(B)**, side view of the E protein molecule.

We analyzed the association between PRNT50 results and differences in the primary sequence of protein E of the studied TBEV strains. It is known that TBEV subtypes differ by certain amino acid residues in protein E (Ecker et al., 1999; Chernokhaeva et al., 2016b). Here, we describe only those positions in the protein E sequence that differ in the studied TBEV strains (Table 5, Figure 2, Supplementary Figure 1).

Vaccine strains Neudoerfl and K23 differ from vaccine strains 205 and Sofjin by the genotype-specific positions 47, 88, 115, 120, 178, 206, 267, 331, 407, 426, 433, 448, and 458. Each vaccine strain also has individual positions that distinguish it from most of the other TBEV strains.

The sera of mice immunized with the vaccines based on Eur strains Neudoerfl and K23 showed significant differences in the PRNT50 against FE strain 205KGG. This finding indicates that in this case, it is not the genotype specificity that plays a decisive role, but other factors. Protein E of strain K23 bears unique substitutions (Asn52Lys, Ala83Thr, and Lys136Arg) that could be related to these differences. On the other hand, position 306 (Met → Thr) is unique for 205KGG. Another strain (178-79) with a substitution in position 306 (Met → Val) was most efficiently neutralized by Encepur compared with the other three vaccines. Residue 306 at the loop between domains I and III is located near the 5-fold symmetry axis of the virion and can play a crucial role in the neutralization by Encepur-induced nAb properties.

FE strain DV936k was less efficiently neutralized by nAbs induced by the Moscow and FSME vaccines than by those induced by EnceVir and Encepur. Unique substitutions in protein E in the Moscow vaccine strain Sofjin, Gln260His and Thr363Ile, can determine the generally lower titers of nAbs induced by this vaccine. The first substitution is located at the interface between protein E, protein M and the membrane, and the second one is located in domain III and is accessible for nAbs. The Neudoerfl strain differs from strain DV936 mostly in subtype signature or variable positions and possesses a unique Val167Ile mutation in the loop contacting the neighboring dimer. The DV936k strain also carries a unique Gly129Glu substitution, and the side chain of this glutamate residue should be oriented inside the protein, thus leading to the molecule destabilization or conformational change coupled with a possible change in the antigenic profile.

EnceVir-induced nAbs showed a low protective efficacy against strain LK-138 in comparison with the Moscow vaccine. The Eur strain LK-138 is particularly special, given the unique Cys60Gly and Glu170Lys mutations. The Cys60Gly substitution destroys a conserved disulfide bond, which can affect the conformational stability of the protein E, and the Glu170Lys mutation changes the residue charge from negative to positive, thus affecting the contact surface with the neighboring E dimer.

## Discussion

Extensive experience in inactivated, concentrated, whole-virion vaccine use (>30 years for the Moscow and FSME vaccines) in TBE-endemic regions (Amicizia et al., 2013) and experiments on laboratory animals (Holzmann et al., 1992; Leonova and Pavlenko, 2009; Fritz et al., 2012; Morozova et al., 2014; Chernokhaeva et al., 2016b) have demonstrated the high protective efficacy of all currently used vaccines in protection against TBE. However, many aspects of the development of post-vaccination immune responses and mechanisms of protective efficacy remain unclear. Research in this area is important for understanding the reasons behind the rare TBE cases among vaccinated subjects and for designing new vaccines providing a prolonged immune response or combined vaccines protecting against several infectious agents.

The aim of this study was to determine which factors are critical for the protective efficacy of the inactivated vaccine. We used 4 TBEV vaccines based on two FE and two Eur TBEV strains and a set of TBEV strains, which were different in time, regions, and sources of isolation, as well as their passage history and phylogenetic lineages (Table 1, Chernokhaeva et al., 2016b). This can affect the spectra of antiviral Abs and the protective efficacy of the vaccines. We used all vaccines at the same dose (0.1 human dose, as recommended by the manufacturer, without additional dilutions). We proceeded from the fact that manufacturers chose a suitable dose and adjuvant to ensure the highest immunogenicity and protection rate.

### Factors affecting the evaluation of the nAb spectrum using PRNT50

The spectrum of nAbs induced by inactivated vaccines depends on the vaccine strain and sometimes also on the recipient (Jarmer et al., 2014; Maikova et al., 2016). Here, we used pooled sera from immunized mice to reduce the influence of individual characteristics of the recipient and allow a detailed assessment of the possible spectrum of induced nAbs.

PRNT50 in the cell culture is a standard method for assessment of the spectrum of antiviral nAbs in the sera. For all vaccines, the highest nAb titers in the sera of mice immunized with a particular vaccine were not against the vaccine strain but to a different one, sometimes even of another subtype. Hence, in the PRNT50, characteristics of a particular virus sample used in the test can play a role along with the antigenic similarity. In a virus suspension, protein E can be found (1) on the surface of virions capable of forming plaques in cell culture, (2) on the surface of non-infectious RNA-containing viral particles, (3) in immature non-infectious virions carrying non-processed prM on their surface, (4) in empty virions lacking the nucleocapsid, and (5) as free protein E (not bound to the virions). All these protein E pools can interact with nAbs and affect the results of the PRNT50. The proportion between these forms of protein E in the viral preparation depends not only on the virus properties but also on the isolation (cell substrate, time of virus harvesting, cytopathic effect, etc.) and storage conditions. To reduce the influence of these virus sample peculiarities on the PRNT50 results, we evaluated the nAb titers in the sera pools against each TBEV strain in the one experiment under standard conditions.

### Analysis of the spectrum of nAbs induced by TBEV vaccines and assessment of the antigenic relationship between the vaccine strains and TBEV strains used in the PRNT50

All vaccines induced nAbs in protective titers against all TBEV strains used in the study after a dual immunization in mice. The highest titers of antiviral nAbs in the sera varied from 1:160 to 1:950. All studied TBE vaccines differed in E protein content. The data presented allow estimating some correlation between E protein content and titers of the induced nAbs. It should be noted that, nevertheless, the content in the Moscow vaccine is 0.5–0.75 μg, which is 2–3 times lower than that in other vaccines; the highest nAb titers after immunization with the Moscow vaccine was 4-fold lower than after immunization with other vaccines. The different E protein contents of the vaccines can possibly affect the spectra of antiviral Abs and protective efficacy. We used all vaccines in the same dose (0.1 human dose) for the mouse immunizations, as they are used for the vaccination of humans according to the manufacturers' instructions.

The breadth of the spectrum of antiviral nAbs induced by vaccines based on different strains was evaluated by the variance between the titers of nAbs against different strains. The vaccines varied by this parameter. The broadest spectrum of nAbs that neutralize TBE strains representing all known subtypes was found in the sera of mice immunized with the Moscow vaccine. This fact indicates that there is no strong correlation between the titers of induced nAbs and the breadth of their spectrum.

The above-described PRNT50 protocol allows evaluating the degree of antigenic similarity between the vaccine strain and the virus used. We analyzed the relationship between the results of PRNT50 and the primary structure of protein E in the vaccine and viruses used in the PRNT50. The vaccine strains that belong to different TBEV subtypes evidently differ by 13 genotype-specific sites in protein E. Each strain also carries specific amino acid residues. The availability of two vaccine strains of the FE and two vaccine strains of the Eur subtypes makes our task considerably easier: if the results of the PRNT50 for two vaccine strains from the same subtype differ, the subtype-specific sites apparently do not play an important role in this case.

The results of the PRNT50 showed that even a single amino acid substitution in protein E of a vaccine strain can affect the spectrum of induced nAbs. In our experiments, this can be seen from the differences in nAb titers against strains 205KGG and DV936 induced by the FSME and Encepur vaccines that belong to the same subtype.

On the other hand, single amino acid substitution in protein E of the TBEV strains used in the PRNT50 can also be of great importance. In our experiments, this was clearly seen for the Encepur vaccine. Neutralizing Abs induced by this vaccine effectively neutralized strain 178-79 and poorly neutralized strain 205KGG that both carry individual substitutions at position 306 of protein E.

### Factors affecting the assessment of the protective efficacy spectrum of the vaccine in experiments on mice

To assess the contribution of antigenic characteristics of the vaccine strain to the efficacy of protection from virulent viruses in mice, we compared the results of *in vivo* and *in vitro* experiments. Our comparison drove us to a conclusion that the vaccines in most cases induced sufficient titers of antibodies to protect the animals from different doses of virulent TBEV strains. Cases in which (despite high titers of nAbs) we observed a low protective efficacy suggest that the difference between the level of protection of immunized animals against various virus strains can be associated not only with a match/mismatch between the spectrum of vaccine-induced antibodies and the antigenic structure of the virus used for the challenge but also with other characteristics of the viruses not related to antigenic structure, e.g., rate and level of viral replication at the early stages of infection; the rate of virus penetration into the cell, i.e., the period when the virions are located outside the cells and are accessible for antibodies; and ability of the virus to modulate the immune response, etc.

In light of this finding, we compared the vaccines by their ability to protect against different TBEV strains in the same experiment under standard conditions. A total of 7 viruses were used, including representatives of the two new distinct phylogenetic lineages. We tested (Tables 3, 4) the Moscow, EnceVir, and FSME vaccines in two different experiments against Eur strain Absettarov; the FSME vaccine was tested twice against Eur strain LK-138; and the Encepur vaccine was studied twice against Sib strain EK-328. The protective effect values were similar. These repetitions showed the reproducibility of our *in vivo* experiments.

The results (Table 4) suggest that almost all vaccines protected the animals from all known virus lineages. However, the protective efficacy of vaccines against some TBEV strains can significantly differ.

The Moscow vaccine based on FE strain Sofjin was tested in 9 experiments against 7 TBEV strains and had a protective effect on more than 80% of the animals (Table 4). The EnceVir vaccine based on FE strain 205 was tested in 4 experiments against 3 strains representing the major TBEV subtypes and showed high efficacy.

The FSME vaccine was tested in 12 experiments (Tables 2, 3) against 10 TBEV strains representing all subtypes and two new phylogenetic lineages. More than 80% of the mice were protected from Eur strains Absettarov and LK-138, FE strains 205 and DV936, Sib strain YuK4/13, and strain 178-79, a TB group representative. This vaccine significantly protected mice from Sib strain EK328 (67%) and from strain 886-84, an MB group representative (47%). However, these rates were lower in comparison with the Moscow vaccine used in the same experiment.

The Encepur vaccine, which was based on Eur strain K23, was tested in 4 experiments against representatives of all 3 TBEV subtypes. The protective effect of this vaccine against strains of the Eur and Sib subtypes was 70 and 80%, respectively. Moreover, Encepur demonstrated a very weak protective effect against FE strain Sofjin.

It can be concluded that phylogenetic relations between a vaccine and a challenge virus are important but are not the only characteristics determining the vaccine protective efficacy against a particular virus, and the strain peculiarities (E protein structure, etc.) can play an important role.

The mapping of variable amino acid residues onto the structure of the E protein did not reveal any general pattern (Figure 2), whereas subtype-specific substitutions and variable positions are mostly localized on the surface of the viral particle or in the regions of the stem and anchor interacting with the ectodomain. Unique point substitutions appear in different regions, with some of them directing their sidechains inside the E protein molecule. Although exposed sidechains may easily influence the recognition of certain epitopes, the effect of buried sidechains is less straightforward. They may be important for dynamic epitope properties. In certain cases, the unique point substitutions can affect the protective efficacy of certain vaccines.

## Conclusion

Immunogenic properties of inactivated vaccines are determined by the titers of induced nAbs and the spectrum of neutralizing Abs. The data presented here show that antigenic cross-reactivity between the vaccine strain and the challenge virus is important, but it was found to not be the sole characteristic determining the protective efficacy.

The protective efficacy of vaccine preparations against different TBEV strains depended on the individual properties of the vaccine strain and the challenge virus rather than on their subtypes.

The neutralization efficiency of nAbs induced by the inactivated vaccine appears to be dependent not only on the presence of nAbs to particular epitopes of the E protein of the challenge virus but also, less directly, on the intrinsic properties of the E protein structure.

## Author contributions

LC, YR, LK, and LR performed the experiments. DO performed the bioinformatics part. LC and DO drafted the manuscript. GK, LK, DO, and LR prepared the final manuscript. GK and MV designed the experiments and overseen the work. All authors reviewed and agreed to the final version of the manuscript.

### Conflict of interest statement

MV worked for Manufacturing Unit of Chumakov FSC R&D IBP RAS – TBE vaccine producer. The remaining authors declare that the research was conducted in the absence of any commercial or financial relationships that could be construed as a potential conflict of interest. The reviewer DB and handling Editor declared their shared affiliation.
