# Supplementary material for: Experimental Evaluation of the Protective Efficacy of Tick-Borne Encephalitis (TBE) Vaccines Based on European and Far-Eastern TBEV Strains in Mice and in Vitro
Source: Front Microbiol. 2018 Jul 16;9:1487. doi: 10.3389/fmicb.2018.01487 (PMC6054986; doi:10.3389/fmicb.2018.01487)
Supplement: Supplementary file 1 [file Image_1.PDF]

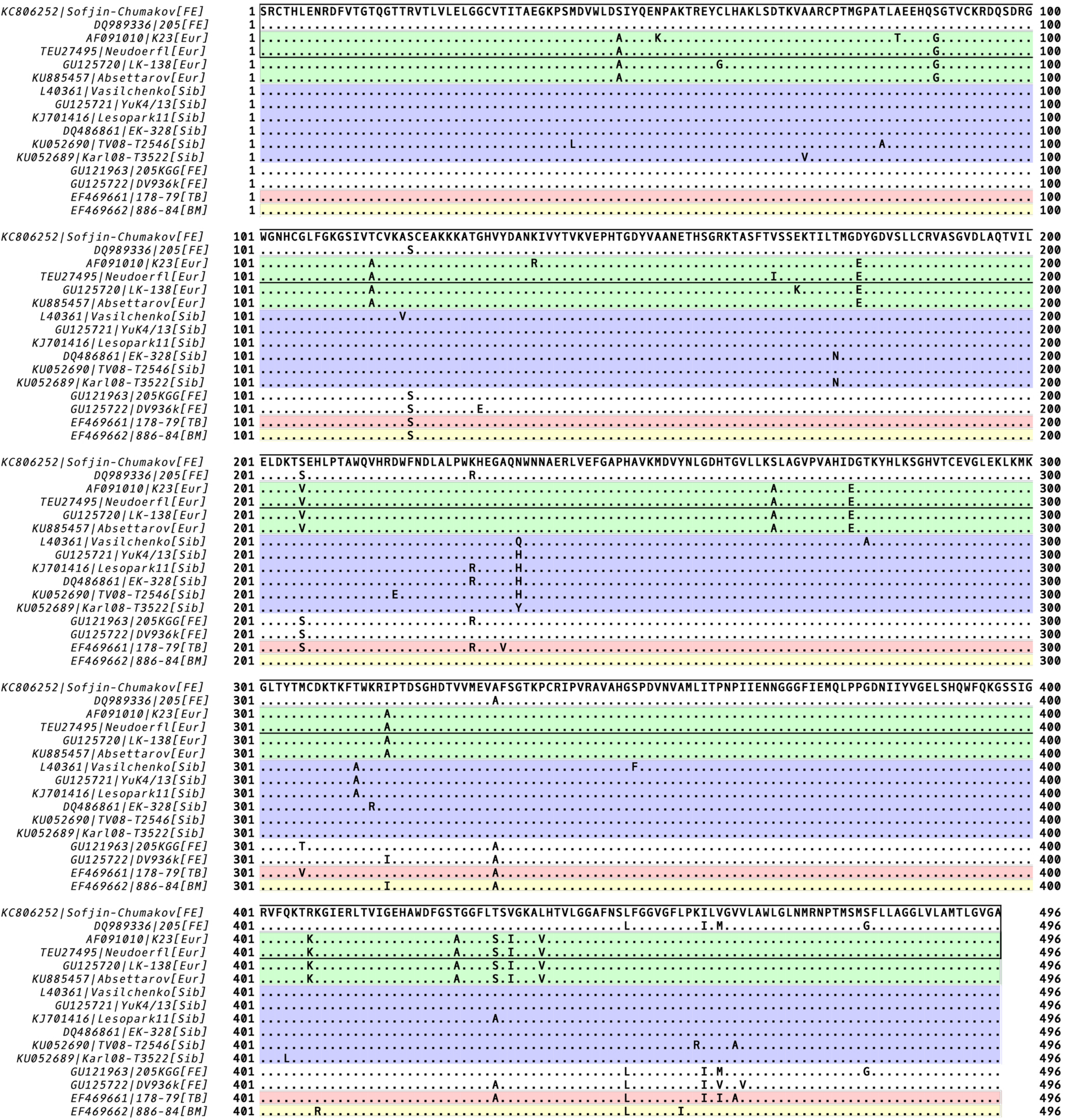

Figure S1 – Alignment of E protein sequences of TBEV strains used in the present study.

Sequence names are written in the form “GenBankID|strain_name[subtype]”.

Residues identical to the first sequence are shown as dots. Vaccine strains are framed.

Background colouring corresponds to subtype: FE, white; Eur, green; Sib, violet; TB, salmon; BM, yellow.
